# The bone marrow pericyte: an orchestrator of vascular niche

**DOI:** 10.2217/rme-2016-0121

**Published:** 2016-11-25

**Authors:** Giuseppe Mangialardi, Andrea Cordaro, Paolo Madeddu

**Affiliations:** 1 Division of Experimental Cardiovascular Medicine, Bristol Heart Institute, University of Bristol, UK

**Keywords:** bone marrow, CD146, endosteal niche, Nestin, perivascular cell, vascular niche

## Abstract

The concept of pericyte has been changing over years. This cell type was believed to possess only a function of trophic support to endothelial cells and to maintain vasculature stabilization. In the last years, the discovery of multipotent ability of perivascular populations led to the concept of vessel/wall niche. Likewise, several perivascular populations have been identified in animal and human bone marrow. In this review, we provide an overview on bone marrow perivascular population, their cross-talk with other niche components, relationship with bone marrow stromal stem cells, and similarities and differences with the perivascular population of the vessel/wall niche. Finally, we focus on the regenerative potential of these cells and the forthcoming challenges related to their use as cell therapy products.

Regenerative medicine is a common term used in identifying the field of research that focuses on employing the techniques in testing the ability of regeneration and restoration in damaged cells, tissues or organs in restoring functionality. This field of medicine is consistent of four distinct fields: cellular therapy, tissue engineering, gene therapy and biomedical engineering. In recent years, application of cellular therapy has gained great attention thanks to a plethora of exciting findings in the fields of stem and progenitor cell research.

Stem cells, as the identifiable members in the field of cellular therapy, are well known for their abilities of self-renewal and direct differentiation. More distinctly, the cohort of embryonic stem cells (ESCs), induced pluripotent stem cells (iPSC) or adult circulating and resident stem cells are well characterized as viable options for their application in the fields of preclinical and translational research [1]. The latter category, adult stem cells, can give rise only to cells of a given germ layer and are thus listed as multipotent. The field of vascular regeneration aims at the restoration of normal vascular structure and function via the formation of new vessel (vasculogenesis) or the sprouting from the pre-existing ones (angiogenesis). Several multipotent cells have been proposed for the therapeutic applications of vascular regeneration in relieving the symptoms of ischemia, prevention of hypoxia-related tissue damage and avoidance of well-known vascular complications such as thrombosis, dissection or capillary leakage. Unfortunately, in spite of promising initial *in vitro* and *ex vivo* results, the adult stem cells failed to deliver an equally positive outcome in clinical studies [2,3]. From this point of view, endothelial progenitor cells (EPCs) offer a paradigm of this development. Characterized for the first time in 1997 by Asahara *et al*. [4], EPCs have shown that they can contribute to neovessel formation, wound healing and intimal re-endothelialization [5]. Unfortunately, a lack of standardization led to mixed results in their potential therapeutical applications [6]. This failure can be attributable to the presence of different isolation protocols, the lack of consensus about a precise antigen characterization, and the lack of a functional assay to define cell role and properties [7]. If a lesson could be learned from the EPC story it is that a scientific consensus is needed in identifying the phenotypic and functional properties of the noted cells for the establishment of a standardized isolation, characterization and culturing protocols for their future applications in clinical trials.

Parallel with the fall of the EPCs, the idea that vascular progenitor cells reside in the vessel wall emerged. Many reports identified similar populations isolated from different tissue with angiogenic potential though without endothelial lineage commitment [8–15]. Those populations can be identified among the broad group of the mural cells. Due to their perivascular position and their multilineage potential, they can be considered the *in situ* equivalent of bone marrow (BM) mesenchymal stromal cells (MSCs) or as perivascular stromal cells (PSCs) (Table 1). In recent years, an incredible number of findings have been gathered about these populations, and the concept of mural cell has evolved accordingly [16]. The BM is the main reservoir of stem and progenitor cells during adulthood. It has received particular attention as the architecture of the tissue is yet to be clearly elucidated. Additionally, in the peripheral vascular wall, different kind of perivascular population, which respond to different functions have been characterized, isolated and expanded, opening a huge debate on vascular progenitor cell hierarchy [17–20].

**Table 1.** **Vascular progenitor populations.**

**Study (year)**	**Tissue of extraction**	**Isolation protocol**	**Markers**	**Ref.**
**Microvascular pericytes**				

Covas *et al*. (2008)	Human retina	Flow cytometry sorting for CD146	NG2^+^, CD146^+^, CD271^+^ and CD140B^+^	[12]

Dellavalle *et al*. (2007)	Human skeletal muscle	Culture adherence selection	ALP^+^, desmin^+^, α-SMA^+^, vimentin^+^ and PDGFRβ^+^	[13]

Crisan *et al*. (2008)	Human fetal skeletal muscle, human fetal pancreas, human placenta, human umbilical cord and other tissues	Flow cytometry sorting for CD146 and ALP	CD146^+^, CD34^-^, CD45^-^ and CD56^-^	[14]

Zimmerlin *et al*. (2010)	Human adipose tissue	Flow cytometry sorting for CD146, CD45 and CD31	CD146^+^, α-SMA^+^, CD90^+^, CD34^-^ and CD31^-^	[21]

**Adventitial cells**				

Campagnolo *et al*. (2010)	Human saphenous vein	Immunomagnetic sorting for CD31 and CD34	CD31^-^, CD34^+^, CD146^-^ and vWf^-^	[8]

Corselli *et al*. (2012)	Human adipose tissue	Flow cytometry for CD146, CD34 and CD31	CD34^+^, CD31^-^, CD146^-^ and CD45^-^	[9]

Avolio *et al*.(2015)	Human neonatal heart	Immunomagnetic sorting for CD31 and CD34	CD44^+^, CD105^+^, CD146^-^, CD34^+^, CD31^-^ and CD45^-^	[15]

Hoshino *et al*. (2008)	Human pulmonary artery	Culture adherence selection	Vimentin^+^, collagen type-1^+^, CD29^+^, CD44^+^ and CD105^+^	[10]

**Intima cells**				

Naito *et al*. (2012)	Murine liver, lung, heart and skeletal muscle	Tissue digestion with collagenase II and dispase	CD31^+^, CD45^-^ and Sca-1^+^	[22]

Fang *et al*. (2012)	Murine lung	Flow cytometry sorting	lin^-^, CD31^+^, CD105^+^, Sca1^+^ and CD117^+^	[23]

Zheng *et al*. (2007)	Human skeletal muscle	Flow cytometry sorting	CD56^+^, CD34^+^ and CD144^+^	[24]

Table recapitulating the progenitor population of the vessel-wall niche. Three main group of progenitors can be identified according to their localization in the vessel layers.

In this review, we aim to provide an overview of the main perivascular players in the BM, how they interact with other BM populations and influence the tissue structure. In addition, we discuss the stem/progenitor features and therapeutic potential of those cells, which could be exploited as a novel product in the regenerative medicine field.

## Pericytes revised

For years, pericytes were believed to be important just for their role in trophic support and stabilization to the vasculature. They share a common basement membrane with endothelial cells (ECs), and both produce basement membrane factors [25]. They are not completely separated from ECs but contact through cell junctions: the peg–socket interaction where pericyte cytoplasmic ‘pegs’ insert into EC invaginations; the gap junctions that allow diffusion of signaling factors; and the adhesion plaques that anchor cells to the basement membrane [26]. The density of pericytes in the body is tissue-specific, and the EC-to-pericyte ratio ranges from 1:1 (in the brain) to 10:1 (in skeletal muscle), while the normal pericyte coverage on the endothelium ranges from 10 to 70%. This distribution appears to have a relationship with the vascular permeability barrier, endothelium turnover and blood pressure [27,28]. There is a continuum of vascular smooth muscle cell/pericyte structure from arterioles to venules [27].

The common idea of pericyte has been revolutionized in the last recent years. In the first instance, aside from microvascular pericyte other perivascular populations have been discovered. If while microvascular pericyte normally resides in the tunica media, adventitial cells have been characterized in the tunica adventitia [8–10,29]. They share a common antigenic profile with microvascular pericytes, although the two populations can be distinguished according to differential expression of some exclusive markers. Both populations have been systematically isolated from different tissues, demonstrating an embryogenic continuity among them. Most importantly, both cells are proven to have a multipotent lineage commitment. Another group of progenitor population has been identified in the tunica intima, even although they cannot be considered proper perivascular cells [23]. These populations are characterized by an endothelial lineage commitment to a different extent. A side population isolated in a murine model showed a greater clonogenic and angiogenic capacity than mature ECs and formed functional vessels *in vivo* [22]. Another study identified the myogenic ECs, a rare subset of myogenic precursor cells that co-expresses myogenic and EC markers (CD56, CD34, CD144) at the microvascular level [24].

The discovery of these populations supported the idea that blood vessels may contain their own multipotent resident population, able to regenerate small and large vessels as well as surrounding tissue. Thus, the idea of a vessel wall niche has become widely accepted [16]. In preclinical studies, those populations have demonstrated a regenerative angiogenic, myogenic, chondrogenic and osteogenic potential [16,30–31].

## BM spatial & functional organization

The BM is a spongy tissue encapsulated within bones involved in hematopoiesis for the production of blood cells in the red marrow of flat and long bones; yellow marrow is found in the medullary cavity and consists of adipocytes. BM is encased in vascularized and innervated bone with trabeculae projecting in the metaphysis. The medullary cavity is lined by endosteum that consists of bone-forming osteoblasts and bone-resorbing osteoclasts [32]. Arteries enter through foramina nutricia and coalesce into venous sinusoids made of a single layer of ECs that act as a conduit to the circulation [33]. In order to mature, hematopoietic stem cells (HSCs) reside in hematopoietic niches. Those are specialized microenviroment which provides the support and signals needed for the differentiation of HSCs into mature cells. The niches relocates during fetal development from yolk sac to aorta–gonad–mesonephros region, then to placenta and fetal liver, and finally to BM, which is the specialized tissue in adult life for hematopoiesis. In the niches different stromal cell and extracellular matrix surround the HSCs in order to regulate their mobilization, differentiation and quiescence [34,35]. The two distinct niches include the endosteal niche, lining the bone surface, and the vascular niche around sinusoids.

### The endosteal niche

HSCs in the endosteal niche exhibit a maturation gradient, with more committed progenitors centrally, and primitive HSCs with greater proliferative potential at the endosteum [36]. Osteoblasts may not maintain HSCs directly but by secreting factors. Transplanted HSCs into irradiated wild-type mice migrated to the endosteum, indicating indirect effects of osteoblasts, as high ionic calcium concentrations attract calcium-sensing receptors on HSCs [37]. HSC maturation is regulated by Notch signaling with osteoblasts, and osteoblasts secrete SCF for HSC self-renewal [38]. The Tie2 receptor binds Ang-1 produced by osteoblasts to maintain HSC quiescence [39,40]. Studies that increased osteoblasts by strontium only found a late increase in HSCs, further suggesting an indirect role [41].

Osteoclasts, which differentiate from precursor cells via RANKL, regulate HSC mobilization, especially under inflammation or hypoxia. RANKL is a type II membrane protein on osteoblasts and Kollet *et al*. found the stimulation of osteoclasts induced the egression of HSCs via CXCR4 and MMP-9 [42]. In addition, SDF-1α and osteopontin were reduced leading to HSC egression. However, RANKL did not stimulate mobilization with impaired osteoclasts under stress stimuli, highlighting a role of osteoclasts in regulating HSC recruitment and homeostasis [43].

Among the diverse group of cells in the endosteum, macrophages also regulate HSCs. Granulocyte colony-stimulating factor suppresses osteoblasts and SDF-1α expression promoting HSC mobilization [44,45]. This mobilization is further enhanced as GCS-F depletes an endosteal macrophage population called ‘osteomacs’, which support osteoblasts [46]. Together, this suggests the involvement of endosteal region in multiple cellular interactions controlling HSCs.

### The vascular niche

HSCs in the vascular niche are associated and influenced by a variety of different cell type. ECs contribute to HSC biology, primarily through the production of angiocrine factors. Akt and p42/44 MAPK are cell-regulating pathways. Akt activation in ECs, both *in vitro* and *in vivo*, promotes HSC expansion and self-renewal through upregulation of factors including IGFBP2, FGF2, BMP4 and DHH, and in parallel the downregulation of HSC-inhibitory factors dickkopf WNT signaling pathway inhibitor 1 and Ang2. MAPK-activated ECs have contrary effects by differentiating HSCs when HSC-stimulating factors are downregulated. However, co-activation of Akt and MAPK ECs increases Notch ligands to prevent exhaustion of HSCs and accelerates differentiation [47]. Thus, the control of Akt and MAPK activation by angiocrine factors can regulate HSC homoeostasis. Akt can be activated by pericyte-derived Ang1 and inhibit EC apoptosis, and is implicated in insulin-stimulated glucose uptake [48]. Along with a paracrine action, ECs can interact in a cell-to-cell contact with HSCs. E-selectin, exclusively expressed by BM ECs, is needed to maintain quiescence in HSCs [49].

**Figure 1.**
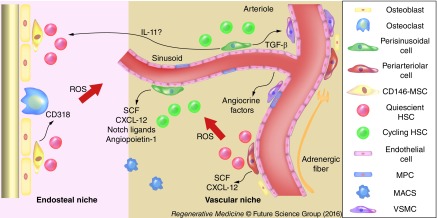
**Spatial organization of bone marrow niches.** Cartoon illustrating the organization of the vascular and endosteal niches and the distribution of the perivascular population. Periarteriolar cells are located atop the intima of the larger vessel and they secrete SCF and CXCL-12 factor in order to maintain HSCs in a quiescent state. Perisinusoidal cells are located around capillaries. They exert a regulation over proliferative HSCs ready to enter the blood stream through the fenestrated BM endothelium. HSCs located in the endosteal niche and around the adventitia of arterioles are less oxygenated and thus less exposed to ROS. This distribution creates an ROS gradient in which dormant HSC localize accordingly. BM: Bone marrow; HSC: Hematopoietic stem cell; ROS: Reactive oxygen species; SCF: Stem cell factor.

The other main regulator of the vascular niche is represented by the heterogeneous group of MSCs. Subsets of different perivascular MSCs have been identified and investigated in the last few years. These cells localize adjacent to marrow vessels and can be identified by different markers besides the typical mesenchymal ones (CD105, CD90, CD73). They can express CD146 [19], CXCL-12 [18], Nestin [50] and Leptin receptor (LepR) (Figure 1) [51]. All these populations have in common the secretion of the factors needed for HSC maintenance: CXCL-12 and SCF. Wild-type perivascular cells synthesize SCF and support hematopoiesis only when implanted into defective S1/S1^d^ murine cells and not separately, suggesting cell–cell contact is required for HSC maintenance [52]. In particular, HSCs need cell-to-cell interaction with perivascular cells as HSCs were depleted in *Sl/Sl^d^* mutant mice, which express the soluble form of SCF but not the membrane-bound one [53]. SCF supply to the niche microenvironment is shared with ECs. In fact, deletion of SCF from LepR^+^ PSCs or ECs depletes HSCs [51], while deletion from osteoblasts, HSCs or Nestin^+^ BM cells showed no effect on HSC population [51]. The other key factor is represented by CXCL-12. One of the first perivascular populations to be identified was indeed the CXCL-12 abundant reticular (CAR) cells in the seminal work from Sugiyama *et al*. [18]. Deletion of CXCL12 from osteoblasts has no effect on HSCs, while deletion of CXCL-12 from osterix-expressing stromal cells, which include CAR cells and osteoblasts, results in constitutive HSC mobilization. CXCL-12 deletion from ECs results in a modest loss of long-term repopulating activity [54].

The HSC niches are not regulated by a single component but by the combination of multiple cells. The nervous system exerts an overall control over the cellular interactions. Circadian noradrenaline release from sympathetic nerves synapsing on perivascular cells regulates the release of SDF-1α and the mobilization of HSCs [55,56]. The nervous system also acts directly on HSCs as CD34^+^ cells express dopamine and β2-adrenergic receptors [57].

## BM pericytes: a heterogeneous & diverse population

As discussed in the paragraph above, the vascular niche is characterized by the presence of different perivascular populations [58]. In recent years, populations with similar antigenic profile have been studied both *in situ* and expanded *ex vivo*. They can be mainly divided in two main groups according to their anatomical position (Table 2). This spatial disposition can remind the organization of the vessel-wall niche. Likewise, a third progenitor population can be identified in the intima of BM vessel (Figure 1).

**Table 2.** **Bone marrow perivascular population.**

**Study (year)**	**Tissue of extraction**	**Isolation protocol**	**Markers**	**Ref.**
**Perisinusoidal cells**				

Sacchetti *et al*. (2007)	Murine BM	Adherence selection and flow cytometry sorting	CD45^-^CD146^+^	[19]

Tormin *et al*. (2011)	Human BM	Colony-forming assay	lin^-^, CD271^+^, CD45^-^ and CD146^+^	[17]

Corselli *et al*. (2013)	Human fetal BM	Flow cytometry sorting for CD146, CD45 and CD34	CD146^+^, LepR^+^, vWF^-^, CD45^-^, CD34^-^ and CXCL-12^+^	[20]

**Periarteriolar cells**				

Mendez-Ferrer *et al*. (2010)	Murine BM	CD45 immunomagnetic depletion in combination with mesensphere assay	CD45^-^, Nestin^+^	[50]

Kunisaki *et al*. (2013)	Murin BM	Colony-forming assay	CD45^-^, Nestin^+^, NG2^+^	[59]

**Intima cells**				

Petrini *et al*. (2009)	Human adult BM	Cell adherence selection	Nestin^+^, CD31^+^, CD105^+^, CD90^-^, CD73^-^, CD271^-^	[60]

Table recapitulating the characterized and isolated perivascular population of the vascular niche in the BM. As in the wall-vessel niche, three main groups of cells can be distinguished.

BM: Bone marrow.

### Perisinusoidal population

CD146 also known as melanoma cell adhesion molecule (MCAM) is normally expressed by pericyte from microvessels and capillaries whereas its expression is lacking in mural cells isolated from tunica adventitia. Among the heterogeneous population of BM MSCs, CD146 in combination with other markers define a perivascular population with a pivotal role in vascular niche maintenance and reconstitution [61].

In 2007, Sacchetti *et al*. were the first to demonstrate the existence of a clonogenic CD146^+^ perivascular fraction of MSCs [19]. In an elegant study, authors demonstrated that the ability to form an *in vivo* heterotopic niche (bone and marrow) was a prerogative of human, nonhematopoietic BM MSCs. In particular, this population strongly expressed marker CD146. However, not all the BM MSCs were able to express this marker but only the colony-forming unit fibroblasts (CFU-F) cultures and their clonal progeny [19]. In particular, CFU-Fs were localized in the CD146^+^/CD45^-^ fraction. These cells show the ability to act as a mural cell in co-culture with ECs. In *in vivo* transplantation, CD146^+^ acquire the same phenotype of Sugiyama CAR cells, suggesting they may be their *in vitro* counterpart [19]. The support to the hematopoietic microenvironment was mainly regulated by the secretion of Ang-1 [19]. In 2011, Tormin *et al*. provided a better characterization of the CD146^+^ MSCs fraction. They demonstrated that CFU-F cultures were mainly enriched for CD271^+^/CD45^-^/CD146^-/low^ and CD271^+^/CD45^-^/CD146^+^ cells, which were both characterized by a typical mesenchymal tri-lineage potential, and both were able to reproduce bone and hematopoietic stroma in *in vivo* transplantation. Interestingly, the expression of CD146 was inversely correlated with hypoxia, providing an explanation why *in situ* CAR express CD146 while bone lining CD271^+^ MSCs do not [17].

Beyond the ability to recreate bone and marrow stroma in orthotopic and heterotopic transplantation, CD146^+^ MSCs play a key role in HSCs maintenance. Corselli *et al*. showed that CD34^-/^CD45^-^/CD146^+^ perivascular cells support stemness in human HSCs *ex vivo*, but unfractionated MSCs and CD146^-^ cells induce differentiation jeopardizing HSCs’ ability to engraft [20]. CD34^-/^CD45^-^/CD146^+^ cells were effective to sustain hematopoiesis from both BM and nonhematopoietic adipose tissue acting via cell-to-cell contact mediated by Notch ligands [20]. Another signaling mechanism involved in HSC maintenance could be related to the Wnt pathway. In a recent study, He *et al*. demonstrated that a BM osteogenic precursor (Nestin^+^, Lepr^+^, Sca-1^+^, CD146^+^) can support long-term HSCs by the secretion of SCF and IL-11, and the inhibition of Wnt signaling in a Notch-independent mechanism [62]. This highlights a ubiquitous ability of pericytes not restricted to the BM.

CD146^+^ and CD146^-/low^ cells demonstrated a similar antigenic profile in different studies. Both populations expressed typical MSCs markers (CD105, CD73, CD90), even though the CD146 fraction could express α-smooth muscle actin at a low level. The difference in CD146 expression did not affect the ability to differentiate into adipocyte, chondrocyte and osteoblasts as MSCs. However, in a recent study Espagnolle *et al*. showed that CD146^+^ but not CD146^-/low^ clones of CFU-Fs are associated with commitment to vascular smooth muscle lineage as demonstrated by the upregulation of CD49α, calponin-1, smooth muscle protein 2α and elastin [63]. Those differences translated into functional properties: CD146^+^ clones were able to secrete more matrix than CD146^-/low^ ones. Interestingly, TGF-β1, a key factor in vascular smooth muscle cell differentiation, upregulated CD146 expression whereas FGF-2, an angiogenic factor, downregulates it [63]. Moreover, CD146^-^ cells in the marrow contain a subpopulation with fibroblast properties and a peculiar pattern of cytokine production (i.e., CD318) [64].

### Periarteriolar population

Along with CD146, perivascular populations in humans and mice have been characterized for the expression of intermediate protein filament nestin. In 2010, Mendez-Ferrer *et al*. identified for the first time a Nestin^+^ population in the perivascular marrow [50]. This population express high levels of CXCL-12 and Ang-1 but lacks endothelial markers such as CD31, vascular endothelial cadherin or CD34 [50]. Interestingly, this population is positive for the β-adrenergic receptor and anatomically associated with catecholaminergic nerve fibers, suggesting they can act as a regulator of HSC trafficking [50]. As MSCs, Nestin^+^ cells retain the ability to differentiate *in vitro* into adipocytes, chondrocytes and osteoblasts, while clonogenicity of MSCs is retained only in the CD45^-^/Nestin^+^ fraction [50]. Nestin^+^ cells have also the ability to maintain the HSC niche. Deletion experiments led to depletion of CD48^-^Lin^-^Sca-1^+^c-kit^+^ (LSK) cells and CD150^+^ CD48^−^ LSK cells in the marrow and relatively increased these populations in the spleen, suggesting Nestin^+^ cells are involved in stem cell trafficking [50].

A 2013 study further investigated the relationship between Nestin expression and functional properties of the the vascular niche. Kunisaki *et al*., using Nestin-GFP mice demonstrated nestin is highly expressed in arterioles (Nes^peri^), while perivascular reticular cells closely related to sinusoids are associated with a dim expression (Nes^retic^) [59]. Even though the latter population was the most abundant in the marrow, Nes^peri^ retained the most clonogenicity in CFU-F assay [59]. Nes^peri^ cells were also associated with tyrosine hydroxylase-positive sympathetic nerves and glial fibrillary acidic protein-positive Schwann cells, similarly to previous studies [50,59]. Using a triple transgene mouse model, authors identified a correlation between spatial localization and functional organization of different perivascular cells: Nes^reti^ expressing LepR are situated near the sinusoids and have a higher proliferation rate; Nes^peri^ are localized near the arterioles, express neural/glial antigen 2 and α-smooth muscle actin, and demonstrate a more quiescent state [59]. The latter population has also the ability to maintain HSCs in a dormant state, while the Nes^retic^ LepR^+^ NG2^+^ population maintains more mature HSCs [59]. In a recent study, Ciuculescu *et al*. demonstrated that the Nestin^+^ population exerts a functional control on HSCs via Rac GTPases. Nestin-Cre-directed excision of Rac1 in Rac3^-/-^ mice reduced Nestin^+^ cells, leading to a drastic decrease of arterioles and conversely an increase in sinusoidal mass. The deletion also encompassed a reduction in long-term HSCs, CFU-Fs activity, and circulating progenitor cells suggesting Nes^peri^ cells are in contact with the main reservoir of quiescent HSCs [65].

### Mesodermal progenitor cells

Among BM Nestin^+^ populations, a different subset of MSCs has been recently isolated. Replacing fetal bovine serum with pooled human serum and thanks to selective culture conditions, Petrini *et al*. characterized a rounded, fried egg-shaped population [60]. These cells were positive for Nestin, CD31 but they lack the expression of MSC markers, CD73, CD90, CD166 and CD271, while retaining the expression for CD105. If supplemented with fetal bovine serum, this population gave rise to an MSC progeny [60]. Interestingly, they were positive for pluripotency-associated transcription factors Oct-4, NANOG and to a lesser extent c-MYC but they lacked typical MSC markers, such as RUNX2 and Sox2 [66]. This population, named mesodermal progenitor cells, is thought to be an early MSCs and, even if the *in situ* localization has not been yet elucidated, it is likely that they reside in the tunica intima of BM vessels, in contact with CAR cells or the Nes^peri^ population. Their positivity for CD31 and Nestin accounts for the hypothesis of a primitive progenitor for endothelial lineages [66,67].

### Similarities & diversity with vascular wall niche

The architecture of the perivascular population in the BM vascular niche could directly recall the one of PSCs in the vessel wall; even if some anatomical differences have to be considered. As described above, the vessel wall niche is constituted of two different populations, a microvascular fraction residing atop the tunica intima of small and large vessels and an adventitial fraction located in the tunica adventitia of larger vessels. In the BM, there is a certain degree of equivalence: the perisinusoidal population is directly in contact with ECs and it expresses CD146 as do microvascular pericytes [17,19]. On the other hand, periarteriolar population lacks the expression of this marker as do the adventitial cells and they reside near vessels of the same caliber [59,65]. Interestingly, the lack of the marker in the adventitial progenitor cell (APC) population could be explained in the same way of BM periarteriole cells: likely, it is due to the fact that periarteriolar cells are the less oxygenated cells in the vessel wall. The production of reactive oxygen species is inversely proportional to hypoxic conditions. Reactive oxygen species are a main regulator of stem cell quiescence [68,69]. Not surprisingly, the more dormant cells are located near the periarteriolar space rather than the more oxygenated sinusoids [59]. Thus, it is hypothesized that APCs can work as a reservoir to reconstitute the other perivascular population. Some findings support this ‘centripetal’ interpretation. Iacobazzi *et al*. demonstrated that APCs have a higher resilience to oxidative stress than ECs, resembling the periarteriolar cells [70]. However, no direct comparison with microvascular pericytes was carried out. Moreover, there are findings that demonstrate *in vitro* expanded APCs can acquire a microvascular pericyte phenotype [9]. Considering all these findings, could the BM vascular and vessel-wall niche be considered as an equivalent system just located in a different district? Certainly, at the moment we are far from having a definitive answer. However, the BM niches preserve some exclusive characteristics when compared with its peripheral counterpart. First, both perisinusoidal and periarteriolar cells have the ability to support and maintain the HSCs compartment [20,59]. It is not clear if this specialization could be explained because of the particular BM microenvironment. Microvascular pericyte isolated from other tissue other than BM showed *in vitro* but not *in vivo* ability to support hematopoiesis [71]. Second, it has been observed that ectopic transplantation of BM perivascular cells *in vivo* reproduces the hematopoietic niche [72,73]. It is thus arguable that when *in vitro* artifacts are taken into account, the perivascular cells from the BM niche can represent a progenitor of other tissue PSCs. Alternatively, they may be derived from a common ancestor.

## BM perivascular cell stemness & regenerative potential

### MSCs & pericytes, parents or siblings?

In recent years, the concept of pericyte has undergone several revisions. Pericytes were primarily identified as mural cells with structural and scaffolding properties, able to promote and then stabilize the vasculature. As a more thorough investigation progressed, pericyte populations have been characterized in different tissues. More interestingly, different kinds of perivascular cells with different functions/properties have been identified in the same vasculature. The family of mural cells was enriched with APCs, microvascular pericytes and so on. All these populations have been linked to BM MSCs, because they share a similar antigenic profile and multilineage potential. However, as for BM MSCs, pericytes have to be considered an heterogeneous population, and a better definition of subpopulations and subsets is needed to elucidate relationships among those cells.

At present, there is an open debate about progenitor hierarchy between MSCs and pericytes, and the *in vitro* characterization brings another level of complexity in this scenario. In fact, the *in vivo* perivascular location combined with the immunocytochemical characterization is widely accepted for bona fide identification. *In vitro*, the expression of pericyte-related markers and supportive position in the formation of capillary-like structures by ECs are considered sufficient to define a population as pericyte [74]. The definition of protocols to isolate pericytes (CD146^+^CD34^-^CD45^-^) and adventitial progenitor cells (CD146^-^CD34^+^CD45^-^) from different tissues and their similarities to MSC group led to the hypothesis that all MSCs could act as pericytes [75]. However, Blocki *et al*. demonstrated that not all the MSCs are able to act as pericytes, but only particular MSC subsets [76].

Recently BM perivascular cells entered the stage in this controversial scenario. The multitude of different populations characterized *in vitro* and *in vivo* in murine and human BM suffers from the lack of standardization as MSCs. Indeed, the International Society for Cell Therapy (ISCT) definition presents different limitations although it has been refined through the years [77]. Some populations as the CXCL-12^+^LepR^+^CD146^+^ (i.e., CAR) could be considered the equivalent of microvascular pericytes (CD34^-^CD146^+^) isolated from other tissues. On the other hand, at the moment Nestin^+^ periarteriolar population could be considered a functional peculiarity of BM tissue. A big question mark remains open also in BM: if those perivascular/pericyte cell can be considered a subset of MSCs or, vice versa, should all the MSCs be enlisted in the perivascular family? The isolation of fibroblast-like CD146^+^ populations with profibrotic properties along with the expression of this marker based upon the hypoxic condition (i.e., perisinusoidal cells express CD146 because they are the most oxygenated) leads us forward for the former hypothesis [78]. Surely, this is an issue that needs to be addressed in order to understand vascular and endosteal niche structure and progenitor cell hierarchy.

**Figure 2.**
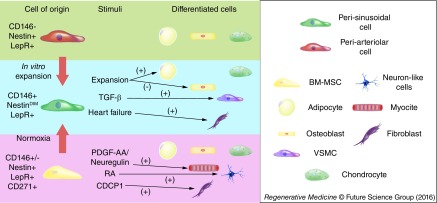
**Bone marrow perivascular cell multipotency.** Recapitulating scheme of multipotent ability demonstrated *in vitro* and/or *in vitro* by BM perivascular cells. CD146^-^ MSCs can acquire CD146 expression after exposure to normoxic condition. Similarly, *in vitro* cultured adventitial cell can phenotypically resemble microvascular pericyte. Perisinusoidal cells can differentiate in the classical tri-lineage of MSCs, although ability to commit to adipocyte fate is lost during *in vitro* expansion. Perisinusoidal cells can also acquire a VSMC-like phenotype after exposure to TGF-β, while a subset of CD146^-^ MSCs has the ability to shift toward a fibroblast-like appearance. Also disease condition as heart failure can push BM CD146^+^ to acquire a fibroblast-like phenotype. BM: Bone marrow; MSC: Mesenchymal stromal cell; RA: Retinoic acid; VSMC: Vascular smooth muscle cell.

Interestingly, along with perisinusoidal cells Tormin *et al*. identified a population of CD271^+^CD146^-^ cells, which retains MSC properties but *in vivo* localizes in the trabecular bone-lining endosteal niche. Another criterion to ascertain the hierarchy between MSCs and PSCs could be plasticity. According to ISCT definition MSCs have to be able to differentiate in at least three different lineages (osteoblast, chondrocyte and adipocyte). Although it was demonstrated that they can differentiate into many more, it is well known that nonclonal MSCs can fail to differentiate in the three classical lineages [79]. Similarly, the population characterized in the BM perivascular group (CAR, Nes^peri^, LepR^+^, among others) can differentiate into the three classical lineages but to a different extent [17,54] (Figure 2). For instance, CAR cells are more prone to a osteoprogenitor path and they tend to lose their adipogenicity through passages [17]. In addition, even if CD146^+^ perivascular cells are shown to support angiogenesis actively *in vitro* and *in vivo*, to date there is no proof that they can commit to an endothelial fate [17]. On the contrary, mesodermal progenitor cells, which lack some of the MSC features, are the most promising cells to achieve *in vivo* endothelial differentiation [60]. Moreover, this population expresses stemness-related transcription factors such as Oct-4 and NANOG, while they are expressed only to a weaker extent in CD146^+^ cells, indicating to they may be the potential mesenchymal ancestor of perisinusoidal and periarteriolar cells [60].

### BM pericyte as regenerative tool

BM progenitor and stem cells have been widely used in animal models and clinical trials in the regenerative medicine field with mixed results. MSCs represented a promising cell therapy opportunity due to the possibility to be isolated from various tissues. Additionally, their immunoprivileged status, multilineage potential and the ability to support angiogenesis in a paracrine fashion make them attractive therapeutically [3]. Despite leading to some encouraging results in animal models, MSCs achieved controversial benefit when cell therapy was translated in humans [3]. The lack of protocol standardization and high cell heterogeneity may be accountable for these drawbacks [3]. BM perivascular cells possess the same advantages of MSCs as they share antigenic and functional profiles, so that they could represent a particular subset of MSCs or the MSC ancestors. Several BM perivascular populations have been extensively characterized using additional markers and/or criteria on top of the ISCT MSC definition. This bypasses one of the major drawbacks of MSCs and offers the opportunity to use a more defined population in regenerative medicine applications. On the other hand, a higher level of antigenic details leads to a smaller population, which raises issues related to expansion, such as loss of multipotent status and occurring senescence [80]. Moreover, as observed in peripheral perivascular cells, biological background can affect cell reparative potential [78,81].

Currently, specific BM pericyte populations have been used in a limited number of studies. Tormin *et al*. demonstrate that lin^-^/CD271^+^/CD45^-^/CD146^+^ cells were able to induce the formation of bone, adipocyte, fibroblasts and capillaries in an immunodeficient mice model [17]. However, due to the heterogeneity of perivascular populations, preliminary studies to investigate and compare the potential of the different subsets are needed. In 2014, Gothard *et al*. isolated different clones from the MSC compartment, comparing their efficacy *in vivo* by subcutaneous implantation in nude mice. They observed that clones expressing Stro-1 and CD146 were the most effective in heterotopic bone formation, while CD105 expressing clones render modest results [82]. In another study, Harkness *et al*. compared flow cytometry sorted MSC-CD146^+^ versus MSC-CD146^-^ isolated from human BM. Both groups were able to form bone and BM elements when implanted subcutaneously in nude mice. Interestingly, the CD146^+^ fraction showed a greater migratory ability both *in vitro* and *in vivo* [83]. Another issue to be addressed is the level of plasticity of these populations in comparison to similar ones isolated from different tissues. In a recent work, Hermann *et al*. compared CD34^-^CD146^+^ MSCs isolated from BM and adipocyte tissue. BM cells showed a better multipotent profile, were superior to their fat tissue equivalent in chondrogenic regenerative applications while cells from the adipose tissue performed better in an osteogenic perspective [84]. This could be explained by the ability of CD146^+^ fraction to respond better to TGF-β stimulation as discussed previously [63]. Additionally, BM-derived perivascular cells were confirmed to promote more efficiently angiogenesis [84].

Apart from the regeneration ability linked to their multipotent lineage commitment, BM pericytes can contribute to the regenerative field favoring the reconstitution of the vascular niche in congenital immunodeficient disease. Mokhtari *et al*. demonstrated that transplanted human CD146^+^ BM cells could increase CXCL-12 production in the large animal fetal recipient, triggering HSC reconstitution [85]. That process could mimic a physiological one. Not surprisingly, Tasso *et al*. observed a recruitment of BM-derived CD146^+^CD105^+^ cells at the site of injection of MSCs [72].

Taken together, these data point out that BM pericytes may reveal themselves as a versatile and useful product in regenerative cell therapy applications.

## Conclusion

The concept of perivascular cells has evolved through years. Initially considered as simply scaffolding cell with the ability to promote and stabilize the vasculature, pericytes are emerging as a heterogeneous multipotent progenitor population. As in other tissues, BM perivascular cells have been studied extensively, and two main populations have been identified: a perisinusoidal and a periarteriolar subtype. Interestingly, both populations play an important role in the maintenance of quiescent and proliferative HSCs through the secretion of different factors (CXCL-12, SCF, IL-11, among others) or directly by cell-to-cell contacts involving Notch ligands in the process. The perisinusoidal subtype is comparable to microvascular pericytes, while the periarteriolar one shares some similarities with ACs. As their peripheral equivalent, those populations showed a multilineage commitment that could be exploited for regenerative purposes. Those populations are intertwined with the heterogeneous group of BM MSCs. It is likely MSCs and BM perivascular cells belong to the same group. Antigenic and functional differences among these populations could reflect the spatial organization in BM architecture.

## Future perspective

Due to their multilineage properties in combination with their ability to support hematopoiesis and angiogenic process, BM perivascular cells can be considered as a novel potential product in the field of regenerative medicine. Their ability to differentiate in osteo- and chondro- progenitors calls for in vitro and in vivo experimentation to pave the way to clinical trials. Moreover, neo-intimal population i.e. mesodermal progenitor cells showed to have the potential to be considered as endothelial progenitor cells. As discussed previously, hematopoietic support ability makes this population well suited as regenerative tool in congenital immunodeficient disease patients.

However, as per any cell-based therapy, there are several issues that need to be addressed. Firstly, basic science studies need to better define all the different perivascular population residing in the bone marrow in order to achieve a scientific consensus about them (i.e. phenotype characterization, population hierarchy). Then, in the perspective of BM PSCs as a regenerative tool, the possible impact of diseases should be investigated. For instance, it is known that condition as diabetes can provide epigenetic modifications which could affect regenerative efficacy. Finally, issues relative to senescence and expandability should be investigated. Those are key steps that need to be taken in order to not undermine the potential of a newly discovered regenerative tool.

**Executive summary**

**Perivascular stem cells**

- Concepts of pericyte have evolved thanks to the discoveries of different perivascular populations with different properties.
- All these populations showed a multipotent ability. Thus it is widely accepted that vessels have their proper stem cell niche, the vessel-wall niche.

**Bone marrow perivascular cells localization & function**

- Bone marrow (BM) is functionally organized in two different niches: the endosteal niche lining of inner part of the bone and the vascular niche, localized around BM vessels.
- In the vascular niche, different perivascular populations have been characterized: perisinusoidal cells that exert maintenance on cycling hematopoietic stem cells ready to egress into vasculature, and periarteriolar cells, which maintain dormant hematopoietic stem cells.

**BM perivascular cells stemness & regenerative potential**

- Both cell types showed multilineage potential (adipogenic, chondrogenic and osteogenic) ability and common markers as mesenchymal stromal cells. Perisinusoidal cells are more prone to differentiate in osteoblasts and less in adipocyte than periarteriolar cells.
- There is an ongoing similarity between perivascular cells of the vessel-wall and BM vascular niche, even though BM cells retain tissue-specific properties.
- Thanks to their better characterized functional and antigenic profile, BM perivascular cells can be exploited as a regenerative tool in particular diseases (i.e., congenital immunodeficient diseases).
